# Declining efficacy of artesunate plus sulphadoxine-pyrimethamine in northeastern India

**DOI:** 10.1186/1475-2875-13-284

**Published:** 2014-07-22

**Authors:** Neelima Mishra, Kamlesh Kaitholia, Bina Srivastava, Naman K Shah, Jai Prakash Narayan, Vas Dev, Sobhan Phookan, Anupkumar R Anvikar, Roma Rana, Ram Suresh Bharti, Gagan Singh Sonal, Akshay Chand Dhariwal, Neena Valecha

**Affiliations:** 1 ECR Division, National Institute of Malaria Research, ICMR Sector 8, Dwarka, New Delhi 110 077, India; 2 National Institute of Malaria Research (Field unit), Guwahati, India; 3 National Vector Borne Disease Control Programme, New Delhi, India

**Keywords:** Plasmodium falciparum, Artesunate + sulphadoxine-pyrimethamine (AS + SP), Artemisinin combination therapy (ACT), *Dihydrofolate reductase* (*dhfr*), *Dihyropteroate synthase* (*dhps*)

## Abstract

**Background:**

Anti-malarial drug resistance in *Plasmodium falciparum* in India has historically travelled from northeast India along the Myanmar border. The treatment policy for *P. falciparum* in the region was, therefore, changed from chloroquine to artesunate (AS) plus sulphadoxine-pyrimethamine (SP) in selected areas in 2005 and in 2008 it became the first-line treatment. Recognizing that resistance to the partner drug can limit the useful life of this combination therapy, routine *in vivo* and molecular monitoring of anti-malarial drug efficacy through sentinel sites was initiated in 2009.

**Methods:**

Between May and October 2012, 190 subjects with acute uncomplicated falciparum malaria were enrolled in therapeutic efficacy studies in the states of Arunachal Pradesh, Tripura, and Mizoram. Clinical and parasitological assessments were conducted over 42 days of follow-up. Multivariate analysis was used to determine risk factors associated with treatment failure. Genotyping was done to distinguish re-infection from recrudescence as well as to determine the prevalence of molecular markers of antifolate resistance among isolates.

**Results:**

A total of 169 patients completed 42 days of follow-up at three sites. The crude and PCR-corrected Kaplan-Meier survival estimates of AS + SP were 60.8% (95% CI: 48.0-71.4) and 76.6% (95% CI: 64.1-85.2) in Gomati, Tripura; 74.6% (95% CI: 62.0-83.6) and 81.7% (95% CI: 69.4-89.5) in Lunglei, Mizoram; and, 59.5% (95% CI: 42.0-73.2) and 82.3% (95% CI: 64.6-91.6) in Changlang, Arunachal Pradesh. Most patients with *P. falciparum* cleared parasitaemia within 24 hours of treatment, but eight, including three patients who failed treatment, remained parasitaemic on day 3. Risk factors associated with treatment failure included age < five years, fever at the time of enrolment and AS under dosing. No adverse events were reported. Presence of *dhfr* plus *dhps* quintuple mutation was observed predominantly in treatment failure samples.

**Conclusion:**

AS + SP treatment failure was widespread in northeast India and exceeded the threshold for changing drug policy. Based on these results, in January 2013 the expert committee of the National Vector Borne Disease Control Programme formulated the first subnational drug policy for India and selected artemether plus lumefantrine as the new first-line treatment in the northeast. Continued monitoring of anti-malarial drug efficacy is essential for effective malaria control.

## Background

Anti-malarial drug resistance, particularly in *Plasmodium falciparum* was a major contributor to global resurgence of malaria in the 20th Century. Southeast Asia (SEA) has been the focus of drug resistance for all anti-malarials with loss of valuable drugs from time to time. Chloroquine resistance in *P. falciparum* malaria was first reported in 1957 in SEA region, later followed by sulphadoxine-pyrimethamine resistance after ten years [1-3]. India shares international boundaries with countries known to be the epicentre for drug resistance. India too experienced increase in falciparum malaria cases with resistance to first-line anti-malarial chloroquine (CQ) in 1973 in northeast India which later spread to other areas in the country [4]. However, resistance to second-line anti-malarial sulphadoxine-pyrimethamine (SP) was also detected in the same area in the country, spreading even more rapidly to other parts of the country. Increasing resistance to CQ and SP forced the national programme to abandon CQ for treatment of *P. falciparum*[5-7], however CQ remains the first-line anti-malarial treatment for *Plasmodium vivax,* another parasite that contributes equally to human malaria in the country.

Since 2005, treatment of uncomplicated *P. falciparum* malaria in the country is based on artemisinin combination therapy (ACT) as per the recommendation of World Health Organization (WHO) [8]. The ACT recommended in the country is artesunate plus SP. Presently, all the five formulations of ACT recommended by WHO except dihydroartemisinin [DHA] + piperaquine, are registered with the Drugs Controller General of India. Prior to introduction of ACT in the country, SP has been used for the treatment of uncomplicated falciparum malaria in India since 1980s as second line [9]. However, resistance to partner drug SP has already been reported in the country [10], which threatens the useful life of this ACT. The mechanism of SP resistance has been well documented compared to other anti-malarials. Point mutations in the *dihydropteroate synthas*e (*dhps*) and *dihydrofolate reductase* (*dhfr*) genes, both coding for essential enzymes in the folate biosynthesis pathway, led to resistance to antifolate drugs [11].

Recent reports on artemisinin resistance in the SEA region [12] also required continuous monitoring of ACT in the country. Artemisinin resistance, defined by delayed parasite clearance following complete treatment, was first reported in SEA along the Thai-Cambodia border in 2006 [12,13]. Recently, parasites with much slower clearance profiles have also been identified in Western Thailand on the border with Myanmar, along the Myanmar-China border and in Vietnam [14-17].

The Indian northeastern region has varied ecological diversity, inaccessible areas where vector control measures as well early treatment and diagnosis facilities are difficult to assess. Malaria control remains a challenge in these areas. ACT was introduced in the study areas in 2007 and since 2009 continuous monitoring of recommended anti-malarials has been done through nationwide sentinel site system. Also, partner drug resistance markers (*dhfr* plus *dhps*) are potential early warning signs; the same were also monitored for the study sites which prompted more focus in these study areas.

This study was part of nationwide sentinel site system initiated in 2009 with the aim to address the continued problem of anti-malarial drug resistance in the country. Continuous monitoring of ACT efficacy was done at the developed sentinel sites. This study reports the findings of the clinical as well as molecular studies at three study sites in the northeastern region.

## Methods

### Study sites

**Figure 1:**
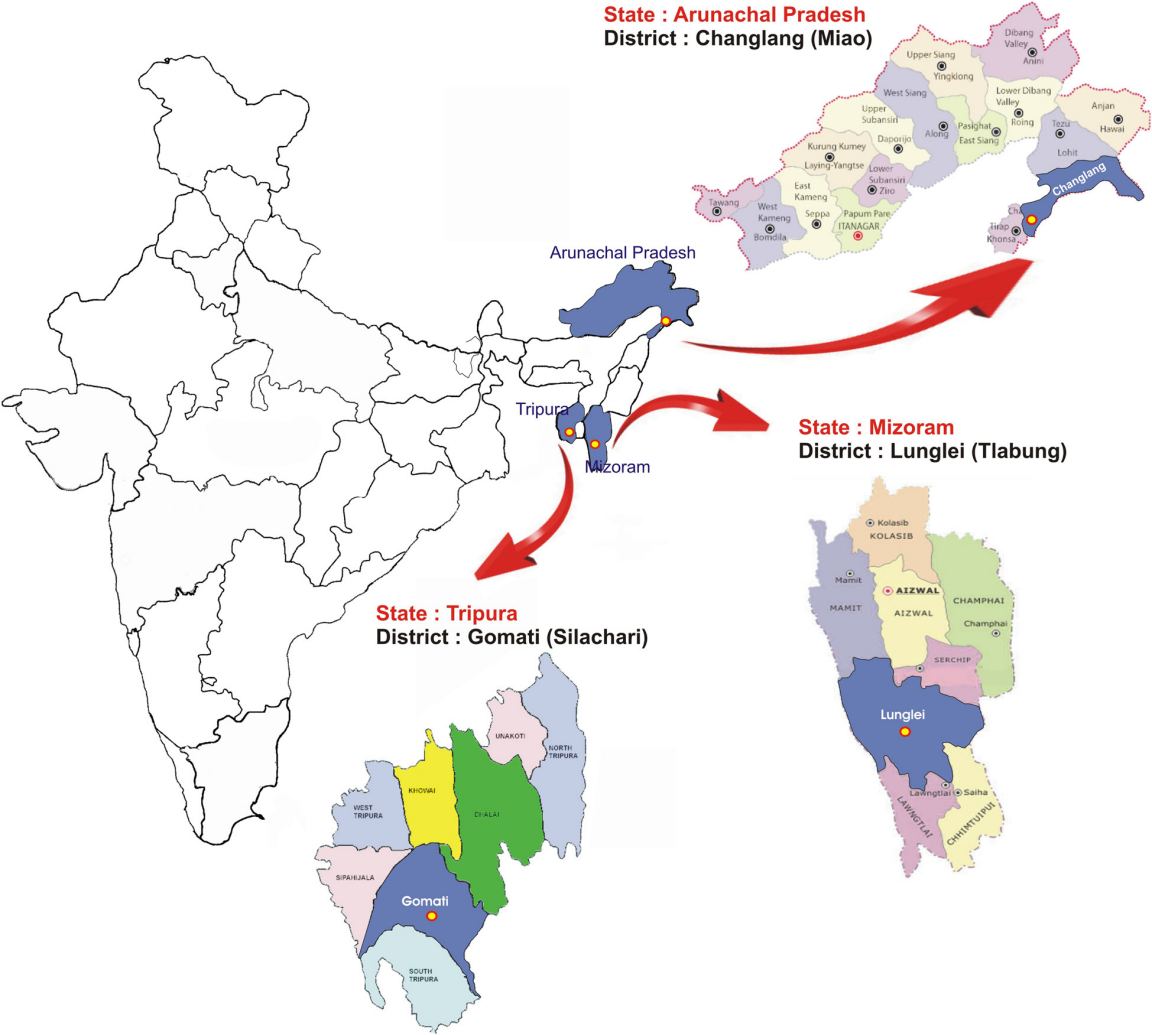
Details of study site from the National Antimalarial Drug Resistance Monitoring System, northeast India, 2012–2013.

The study was conducted from May to August 2012 in Mizoram (district Lunglei, Tlabung Sub divisional hospital); from August to September 2012 in Tripura (district Gomati, Silachari Primary Health Centre (PHC)); from September to October 2012 in Arunachal Pradesh (district Changlang, PHC Miao). The study sites (Figure 1), which include three different regions of northeastern India representing different epidemiological situation of malaria due to international borders, are described below.

### Mizoram

Lunglei district in the state of Mizoram presents ideal ecological conditions for malaria transmission with undulating uplands intersected by forested hills, rocky streams and jhum cultivation land. The area is characterized by a tropical, humid climate with cool summer and cold winter and heavy rainfall during May to late September with an average annual rainfall of 3,006 mm under the influence of southwest monsoon, and the mean annual temperature ranges between 20 and 26°C. The state contributes 0.8% to the country’s malaria burden [18]. *Plasmodium falciparum* accounts for 80 to 90% of the total malaria cases and the proportion of *P. falciparum* has increased from 83 to 96% over the last five years in the study district. The study area of Tlabung subdivisional hospital in Lunglei district is highly endemic for malaria, characterized by year-round malaria transmission.

### Tripura

The state contributes 1.3% to the country’s malaria burden [18]. The peak malaria season is from August to November. The study districts are highly endemic, with a high proportion of *P. falciparum* (87-91%) cases.

Tripura is one of the states in the northeastern region which shares a long international border with Bangladesh. The hilly and undulating terrain and the movement of people across the border have led to persistence of malaria in villages near the border. The regions adjacent to the Indo-Bangladesh border are mostly covered with thick forests and have poor communication and health infrastructure. *Plasmodium falciparum* is a major malaria parasite in this region, causing 70 to 90% of malaria infections. The hot and humid climate in the region is ideal for survival and multiplication of malaria vectors [19].

### Arunachal Pradesh

Changlang district in the state of Arunachal Pradesh is bounded by Assam and Arunachal Pradesh in the north and Myanmar in the southeast. The state contributes 1.1% of the country’s malaria burden [18]. The capital town of Miao, in Changlang district, was selected as the study site where the percentage of *P. falciparum* has increased from 56 to 64% in the last five years.

### Study design

This was an open-label, one-arm prospective study of clinical and parasitological responses after administration of ACT treatment and WHO protocol for *in vivo* monitoring was followed [20].

### Ethical considerations

Informed, written consent was obtained from enrolled adults and from a legal guardian of each child enrolled. The study protocol was approved by the Institutional Ethics Committee of the National Institute of Malaria Research (NIMR) in New Delhi. These studies were part of monitoring and surveillance studies jointly carried out by NIMR and National Vector Borne Disease Control Programme (NVBDCP) with added quality assurance, monitoring and molecular markers.

### Study population and sample size

Adults and children over 6 months presenting with fever (axillary temperature ≥ 37.5°C) at visit or a history of fever for the preceding 24 hours were included in the study. Other criteria for inclusion were mono-infection with *P. falciparum* with parasitaemia between 500 and 100,000 asexual parasites/μl blood, absence of other febrile conditions and informed consent. Patients having severe malnutrition as per WHO guidelines, severe malaria or danger signs and inability to come for follow-up visits were excluded from the study.

### Treatment and follow-up

Patients with uncomplicated *P. falciparum* received artesunate (AS) + SP (AS 4 mg/kg for three days plus SP 25/1.25 mg/kg single dose on day 0) and primaquine (PQ) (0.75 mg/kg) on last day of treatment. The treatment was directly observed on all three days and used quality assured drugs through state government supply which consisted of manufacturers (site name in brackets): Medicamen Biotech Ltd, India (Lunglei), Medico Remedies Pvt Ltd, India (Gomati), ZEST Pharma, India (Changlang). These drugs are supplied in the trade name of ‘Antimalarial combi blister pack’. All drugs were used within their expiry period and batch number and expiry dates were recorded in each case record form (CRF). The dosing was based on age with five categories of combi blister packs with age category < one year, one to four years, five to eight years, nine to 14 years and >15 years (adult pack). The study followed the standard WHO protocol for assessment of therapeutic efficacy of anti-malarial drugs for uncomplicated falciparum malaria for moderate transmission [20]. On enrolment day (day 0), upon fulfilment of all inclusion criteria, consent was obtained from the patients to participate in the study. A brief history was recorded and clinical was done. A pretreatment blood sample was collected from each eligible patient and used to make thick and thin smears and dried blood spots on filter paper. All medicines were given under direct supervision. Patients were observed for a few minutes after administering the study drug to ensure that they did not vomit. Patients who vomited the dose within 30 minutes were retreated with the same drug and dose. Follow-ups were scheduled for days 1, 2, 3, 7, 14, 21, 28, 35, and 42. On each of these days, clinical and parasitological assessments were performed. Blood samples were obtained on filter papers for genotyping and molecular studies on day 0 and every other day of follow-up. Polymerase chain reaction (PCR) genotyping was done for distinguishing recrudescence from new infections by comparing *msp1*, *msp2* (merozoite surface protein) and *glurp* (glutamate rich protein) gene loci of pre- and post-treatment sample pairs. The outcome of treatment with PCR correction was based on the number of true recrudescence excluding cases of novel infections. In addition, *pfdhfr* and *pfdhps* mutations for partner drug resistance were used as molecular markers to analyse different samples.

### Laboratory methods

Thick and thin blood films were collected and stained with Giemsa. Slides were examined on day 0 by experienced microscopist(s) for species identification and quantification of parasites. Slides were also prepared on days 1, 2, 3, 7, 14, 21, 28, 35, and 42 to determine asexual and sexual parasite density by counting the number of parasites against 200 white blood cells (WBCs), and were expressed assuming WBC count to be 8,000/microlitre. A slide was considered negative when counting 1,000 WBC in thick smear did not show asexual parasites. All the slides were cross-checked at NIMR, Delhi.

Paired blood samples of patients collected on day 0 and the day of recurrent parasitaemia (14–42 days) were analysed sequentially starting with the highest discriminatory marker, *msp2* or *glurp*. The third marker analysed was *msp1* to differentiate recrudescence from new infections [21]. A new infection is a subsequent occurring parasitaemia in which all the alleles in parasites from the post-treatment sample are different from those in the admission sample, for one or more loci tested. In a ‘recrudescence’ at least one allele at each locus is common to both paired samples [21]. In addition, SP resistance-associated markers, namely mutations at codon 51, 59, 108, 164 of *pfdhfr* gene and *pfdhps* gene at codon 436, 437, 540, 581, 613 were also analysed in the day 0 samples.

Genomic DNA was isolated from blood spots using QIAamp DNA minikit, Germany. Genotyping PCR assays were carried out following the protocols reported earlier [22]. Separate nested PCR reactions were performed for the three allelic families of *msp1* (MAD20, K1 and RO33), two allelic families of *msp2* (Fc 27 and Ic) and *glurp. Pfdhfr* and *pfdhps* gene products were PCR amplified using earlier reported methods [23] and then digested using restriction enzymes for *dhfr* and *dhps* gene. Applied Biosystem thermocycler was used for all PCR amplification reactions. Digested PCR product (5–8 microlitre) was analysed on 1.5% agarose gel containing ethidium bromide (0.5 μg/ml) and 0.5X TBE running buffer (pH 8.0). PCR products were visualized under UV transilluminator (280 nm) and digitally captured with the help of gel documentation system (Alpha Imager EP). Molecular sizes of PCR fragments were calculated using gene tool (Alpha Inotech, version 3.0.3.0).

The case record forms were completed for each patient and all the clinical and parasitological data from days 0 to 42 were recorded. The data were entered in WHO software [20] and both per protocol and Kaplan Meier analysis were performed to classify response as early treatment failure (ETF), late treatment failure (LTF) and adequate clinical and parasitological response (ACPR). In the secondary analysis, patients were withdrawn or censored, if classified as new infection by PCR or if PCR was missing.

### Rescue medication

All the patients with ETF or LTF were treated with oral quinine in standard dose of 10 mg/kg body weight, three times for seven days. CRFs were used to capture adverse events, if any.

### Data analysis

Patient and demographic variables that could be associated with treatment failure were investigated. Using the body weights recorded on day 0, the doses of AS given to patients with *P. falciparum* were categorized as being ≥ 87.5%, < 87.5 to 75% and < 75% of the recommended dose per kg. The parasite clearance time (PCT) was recorded in CRF till day 2 of treatment, although slides were made on day 3 as well. However the delayed PCT (PCT ≥ 72 hours) was calculated on day 3 based on the mean PCT up to day 2 ± SD. Kaplan-Meier survival analyses of treatment failure and parasite clearance were conducted with and without the results of the PCR-based identification of the parasitaemia that resulted from post-treatment re-infection. Log-risk models were used to evaluate the multivariate associations observed between risk factors and endpoints. Multivariate analysis was based on full models that included age, sex, fever at enrolment, level of parasitaemia at enrolment, infection with a parasite that harboured any of the investigated mutations in *dhfr* or *dhps,* and AS dose, as well as the interactions between age and AS dose, age and level of parasitaemia on enrolment, and between presence of mutations and level of parasitaemia on enrolment. A complete case (per protocol) analysis was performed. A strategy of backward elimination was followed in which p-value of < 0.10 and < 0.15, respectively, were used as the cut-offs in eliminating individual factors and interaction terms as statistically significant risk factors for treatment failures. All data analysis was performed using version 7.2 of a software package developed by WHO’s Global Malaria Programme for evaluating therapeutic efficacy or SPSS version 14.

## Results

**Figure 2:**
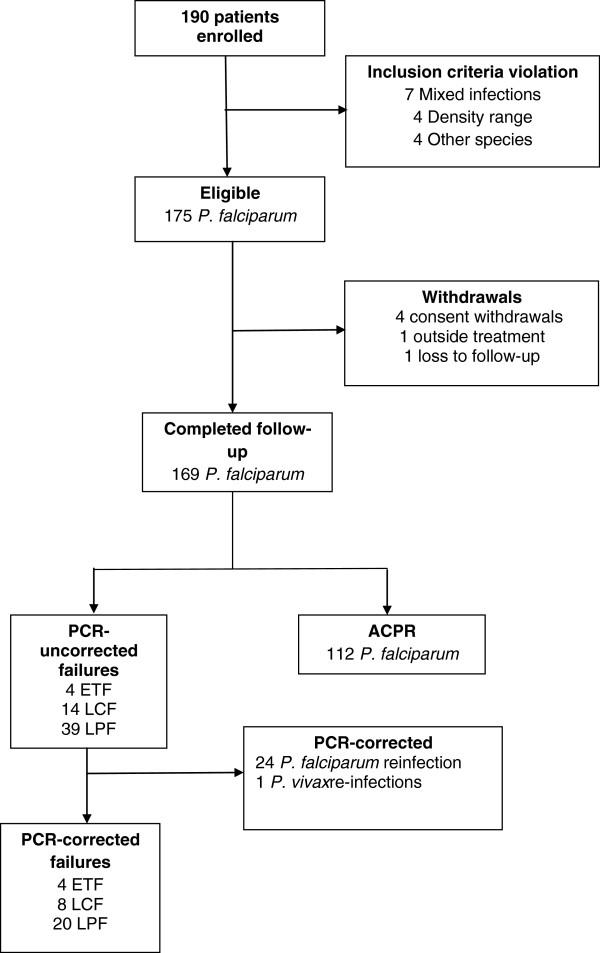
**Patient cohort from the National Antimalarial Drug Resistance Monitoring System, northeast India, 2012–2013.** Abbreviations: ACPR, adequate clinical and parasitological response; ETF, early treatment failure; LCF, late clinical failure; LPF, late parasitological failure; PCR, polymerase chain reaction.

A total of 190 patients with uncomplicated *P. falciparum* receiving AS + SP treatment on first three days and PQ on last day of treatment were enrolled from Lunglei district in Mizoram (71), Gomati district in Tripura (77) and Changlang district in Arunachal Pradesh (42), the three far- flung regions of northeast India. Fifteen patients were withdrawn after cross-checking due to mix infection, presence of other species, or out of range parasitaemia at the time of enrolment. Thus, 175 patients were found to be eligible, 169 (96.6%) of whom completed the 42 days of follow-up (Figure 2).

Almost half of the patients were of either sex and were febrile at the time of enrolment (Table 1) at two sites except Lunglei. The intake of anti-malarial drug in the previous week was rare. High gametocyte carriage was observed in Changlang followed by Gomati and Lunglei districts. The combined point mutation in *dhfr-dhps* gene, indicating partner drug resistance was observed at all the three sites with majority of isolates showing multiple mutations.

**Table 1:** Clinical and demographic characteristics of eligible patients in studies of the National Antimalarial Drug Resistance Monitoring System, northeast India, 2012-13

**Study site**	**Lunglei (Mizoram)**	**Gomati (Tripura)**	**Changlang (Arunachal Pradesh)**
Characteristic	(N = 66)	(N = 72)	(N = 37)
Sex (no (%))			
Male	39 (59.1)	39 (54.2)	22 (59.5)
Female	27 (40.9)	33 (45.8)	15 (40.5)
Age category (no (%))			
< 5 yr	6 (9.1)	24 (33.3)	7 (18.9)
5-15 yr	26 (39.4)	30 (41.7)	18 (48.6)
Adult	34 (51.5)	18 (25.0)	12 (32.4)
Temperature (˚C)			
Mean ± SD	36.3 ± 1.3	38.1 ± 0.4	38.2 ± 0.5
Range	36.2-39.3	37.5 - 39.2	(37.5 - 39.1)
Febrile (≥ 37.5)			
Yes (no (%))	13 (19.7)	72 (100.0)	37 (100.0)
No (no (%))	53 (80.3)	-	-
Parasite count (no/μl)^a^			
Mean ± SD	18899.3 ± 23979.5	18507.6 ± 20937.3	31186.5 ± 27451.6
< 5,000	22 (33.3)	26 (36.1)	7 (18.9)
5,000-50,000	36 (54.5)	37 (51.4)	20 (54.1)
≥ 50,000	8 (12.1)	9 (12.5)	10 (27.0)
Gametocytes on day 0			
(no (%))	3 (4.2)	17 (23.9)	12 (32.4)
Number of mutations			
0-2	6 (9.1)	3 (4.2)	1 (2.7)
2-4	4 (6.1)	5 (6.9)	2 (5.4)
≥ 5	56 (84.8)	64 (88.9)	34 (91.9)

^a^At enrolment, before treatment.

For calculating the primary and secondary endpoints of the 169 patients infected with *P. falciparum* who completed 42 days of follow-up, 57 patients were initially categorized as treatment failures. However, when parasites in paired dried blood spots from these 57 patients were genotyped by PCR, only 32 (56%) of the patients were confirmed as treatment failure, 24 were found to have *P. falciparum* re-infection and one had *P. vivax* infection on day 21. These 32 patients who were confirmed to have failed AS + SP treatment came from Lunglei (11), Gomati (15) and Changlang (six) (Table 2). Four ETF occurred in Gomati (Tripura); two patients had parasitaemia and fever on day 3 while two patients had parasitaemia on day 2 (parasitaemia on day 2 was > 25% of day 0). The crude and PCR-corrected Kaplan-Meier survival estimates were 60.8% (95% CI: 48.0-71.4) and 76.6% (95% CI: 64.1-85.2) for Gomati (Tripura); 74.6% (95% CI: 62.0-83.6) and 81.7% (95% CI: 69.4-89.5) for Lunglei, Mizoram; and 59.5% (95% CI: 42.0-73.2) and 82.3% (95% CI: 64.6-91.6) for Changlang, Arunachal Pradesh, respectively. In general, the *P. falciparum* patients treated with AS + SP cleared their parasitaemia rapidly, 52.1% (88 of 169) in < 24 hours, 35.5% (60 of 169) in intervals of 24 to <48 hours, 8.8% (15 of 169) in 48 to < 72 hours and 4.7% (eight of 169) of the patients in ≥ 72 hours, respectively (Table 2). The highest number of patients clearing parasitaemia within <24 hours was in Lunglei, Mizoram. Even the LTF patients cleared their parasitaemia rapidly; 14.8% (13 of 88), 16.7% (ten of 60), 40% (six of 15) and 37.5% (three of eight) of the patients who showed parasite clearance intervals of < 24 hours, 24 to < 48 hours, 48 to < 72 hours and ≥ 72 hours, respectively. Six of the patients from Gomati, Tripura and two from Changlang, Arunachal Pradesh did not clear their parasitaemia within 72 hours (Table 2). Three of them were identified as treatment failures (including two ETF and one late parasitological failure), two were re-infection whereas the three achieved complete cure. However, the proportion of patients remaining parasite positive at day 3 in these studies was much below the cut-off prescribed by WHO. No adverse effect was noted at any site during the 42 days’ follow-up.

**Table 2:** Site-wise results of therapeutic efficacy and parasite clearance in studies of the National Antimalarial Drug Resistance Monitoring System, northeast India, 2012-2013

				**Therapeutic efficacy**										**PCT (hours)**			
**State/UT**	**District/city**	**Drug**	**n**	**ACPR**^**a**^	**ETF**^**b**^	**LCF**^**c**^	**LPF**^**d**^	**LFU**	**WTH**	**PV**	PF*	**Surv**	**95% CI**	**< 24**	**24- < 48**	**48- < 72**	**≥ 72**
Tripura	Gomati	ASP	77	43	4	3	8	0	8	1	10	74.1	61.0, 84.7	25	26	12	6
Mizoram	Lunglei	ASP	71	47	0	3	8	1	7	0	5	81.0	68.6, 90.1	47	15	1	0
Arunachal Pradesh	Changlang	ASP	42	22	0	2	4	0	5	0	9	78.6	59.0, 91.7	15	18	2	2

*Abbreviations*: *ACPR* adequate clinical and parasitological response, *ETF* early treatment failure, *LCF*, late clinical failure, *LPF* late parasitological failure, *LFU* loss to follow-up, *WTH* withdrawal, *PCT* parasite clearance time, *PF P. falciparum re-infection, *PV* protocol violation.

^a^Absence of parasitaemia on day 28 without previous criteria for failure.

^b^Marked by the following, alone or in combination: danger signs for severe malaria on day 1, 2 or 3 in the presence of parasitaemia; parasitaemia level on day 2 higher than on day 0; parasitaemia level on day 3 more than 25% higher than on day 0; parasitaemia on day 3 plus fever.

^c^Danger signs for sever malaria and/or fever plus parasitaemia from day 4 to day 28.

^d^Parasitaemia from day 7 to day 28 even if patient afebrile.

The study compared the mean parasitaemia at enrolment, dose of AS or any adverse event reported between patients who had parasitaemia ≥72 hours with those who cleared parasite within 48 hours. No significant difference was observed between the two groups. However, host immunity had a definite role as six out of eight patients were < five years of age.

In multivariate analysis, younger age category (< five years) relative to adults 15 years or older and low administered dose of AS (< 3 mg/kg, 3–3.5 mg/kg) relative to 3.5 mg/kg or more were associated with higher risk. The risk of failure increased as the dose of AS decreased. However, optimal or higher dose of SP was observed in 90.6% patients. Fever at enrolment was positively and significantly associated with treatment failures. Also, relative risk was highest with the presence of triple or quadruples mutation (Table 3). There were insufficient observations to determine the risk factors for parasite clearance interval of ≥ 72 hours.

**Table 3:** **Risk of *Plasmodium falciparum *** **treatment failure among patients in studies of the National Antimalarial Drug Resistance Monitoring System, northeast India, 2012-2013**

**Predictor**	**Value**	**Fail**^**a**^	**ACPR**	**Risk**	**RR**	**95% CI**
Artesunate dose (mg/kg)	≥ 3.5	24	84	0.222		
	3.0-3.5	5	21	0.192	0.64	0.19, 2.19
	< 3.0	3	7	0.300	1.35	0.49, 3.71
Age	< 5	13	18	0.419	2.17	1.11, 2.46
(years)	5-15	11	46	0.193	1.35	0.58, 3.11
	≥ 15	8	48	0.143	--	
Parasite count	< 5000	6	38	0.136	0.65	0.28, 1.53
(/μL)	5000 - 50000	17	64	0.210	0.44	0.23, 0.84
	≥ 50000	9	10	0.474	--	
^#^Antifolate	Wild	0	0	0.000	--	
mutations	1-2	0	6	0.000	--	
	3-4	5	11	0.313	1.48	0.65, 3.34
	5-6	22	82	0.212	0.68	0.29, 1.53
	7-8	5	11	0.313	--	
Fever	Yes	25	73	0.255	1.68	0.78, 3.59
(≥ 37.5°C)	No	7	39	0.152	--	
Previous	Yes/unknown	17	69	0.198	0.76	0.42, 1.41
Drug intake	No	15	43	0.259	--	

*Abbreviations*: *ACPR* adequate clinical and parasitological response, *RR* relative Risk, *CI* confidence interval, ^#^*dhfr + dhps.*

^*a*^25 patients were excluded from the analysis as confirmed by PCR.

Gametocytes on day 0 at the enrolment were reported in 20.1% patients with highest number in Changlang followed by Gomati. There was increase in the number of patients carrying gametocytes on day 7 at Gomati, Tripura (Table 4). The presence of gametocyte was observed in 2.3% patients as late as day 42 although radical treatment with single dose of PQ (0.75 mg/kg) was given on last day of treatment. PQ use is however contra-indicated in pregnant women and children aged < one year.

**Table 4:** Gametocyte carriage detected by microscopy in patients

**S No**	**State**	**District**	**Gametocytes/μl (%)**						
			**D0**	**D7**	**D14**	**D21**	**D28**	**D35**	**D42**
1	Tripura	Gomati	17/69 (24.6)	22/69 (31.9)	20/69 (29.0)	14/69 (20.3)	6/69 (8.7)	-	3/69 (4.3)
2	Mizoram	Lunglei	1/63 (1.6)	2/63 (3.2)	0/63 (0)	0/63 (0)	1/63 (1.6)	0/63 (0)	0/63 (0)
3	Arunachal Pradesh	Changlang	12/37 (32.4)	0/37 (0)	1/37 (2.7)	0/37 (0)	0/37 (0)	0/37 (0)	1/37 (2.7)

Out of the 190 isolates, 155 could be successfully genotyped for *dhfr* and *dhps* mutation analysis. In *dhfr,* double (n = 71) and triple mutations (n = 69) were common. The most frequent haplotype was double mutant 108/59 followed by triple mutant 108/59/51. Only five isolates were single mutant while three were found to have quadruple mutation (5.2%).

In *dhps*, triple (n = 75) and quadruple mutations (n = 35) were common. Mutations in codon 437 and 540 were most frequent and the most frequent haplotype was triple mutant 540/437/436 followed by quadruple mutant 581/540/437/436 (Table 5).

**Table 5:** **Molecular markers of anti-folate resistance in *Plasmodium falciparum *** **isolates collected through the National Antimalarial Drug Resistance Monitoring System, northeast India, 2012-2013**

	***dhfr *** **(N = 155)**		***dhps *** **(N = 155)**	
**Mutations**	**n**	**Haplotype**	**n**	**Haplotype**
Quadruple	5	**I**51/**R**59/**N**108/**L**164	1	**A**436/**G**437/**E**540/A581/**T**613
			35	**A**436/**G**437/**E**540/**G**581/A613
Triple	69	**I**51/**R**59/**N**108/I164	75	**A**436/**G**437/**E**540/A581/A613
	7	N51/**R**59/**N**108/**L**164	6	**A**436/**G**437/K540/**G**581/A613
			5	S436/**G**437/**E**540/**G**581/A613
Double	71	N51/**R**59/**N**108/I164	11	S436/**G**437/**E**540/A581/A613
			1	**A**436/A437/**E**540/A581/A613
			2	S436/A437/**E**540/**G**581/A613
			9	S436/**G**437/K540/**G**581/A613
Single	3	N51/C59/**N**108/I164	1	S436/A437/**E**540/A581/A613
			2	S436/**G**437/K540/A581/A613
			1	S436/A437/K540/**G**581/A613
Wild			6	S436/A437/K540/A581/A613

*Mutations in bold.

Among the 32 PCR-corrected treatment failures, two samples were not amplified, one was *dhfr* quadruple mutant, 21 were triple mutants, seven were double mutants and one was single mutant. However, seven samples were *dhps* quadruple mutant, 14 triple mutants, eight were double, and one was single mutant.

Interestingly, *pfdhfr* L164 mutation has been observed in three out of 11 treatment failures in Lunglei, two out of 15 treatment failures in Gomati and in Changlang district. Earlier, *pfdhfr* L164 mutation has been found to be associated with higher level of SP resistance in addition to decreased efficacy of chloroguanil/dapsone. Also *dhps***T**_613_, **G**_581_ and **A**_436_ have also been observed at all the three sites, majority in ACPR samples except one **G**_581_*dhps* mutation in treatment failure sample out of the total four failures in Changlang district.

## Discussion

The sentinel site monitoring studies at three sites in northeastern region indicate that the efficacy of the ACT (i.e., AS + SP) recommended for *P. falciparum* malaria was declining. The treatment failure rates above the 10% threshold warranted a change of drug policy in the region. These treatment failures were mostly LTF with molecular markers for partner drug resistance showing triple or quadruple mutation in *dhfr* and *dhps* genes. Four ETF were observed at Tripura. These patients received optimal dose of AS and SP. As per WHO definition of suspected artemisinin resistance, increased parasite clearance as evidenced by > 10% of cases with parasites detectable on day 3 following treatment with an ACT warrants studies to confirm presence of artemisinin resistance. However, the proportion of patients remaining parasite positive at day 3 in these studies was much below the cut-off prescribed by WHO**.** In addition, artemisinin efficacy studies are being undertaken at two of these sites to confirm the efficacy of artemisinin in the region. It is worth mentioning that three out of four patients were below the age of five years and had higher frequency of quintuple mutation as compared to patients with ACPR, confirming the role of host immunity.

This study provides the first report on the emerging treatment failure of AS + SP in remote areas of the country, including areas directly across the border from Bangladesh and Myanmar where artemisinin resistance has been confirmed recently. Treatment failure to this ACT is now evident at all the three study sites of northeast India. The failure of AS + SP was likely as the molecular marker of partner drug resistance showed increasing trend since 2009 in the nationwide sentinel site monitoring where 20% random samples were assayed to monitor drug resistance at molecular level. The trend showed increase from single to double mutation in majority of the samples. These studies were part of nationwide sentinel site monitoring system, where 15 sentinel sites are being monitored across the country to ascertain the efficacy of recommended anti-malarials against the predominant species of Plasmodium.

Based on the results of this study, the expert committee of the NVBDCP created the first subnational drug policy for India and selected artemether plus lumefantrine as the new first-line treatment in the northeast [24].

ACT treatment failure > 10% is the cost-effective threshold and requires change of treatment policy for malaria along with continuous monitoring in adjacent areas [20]. In the present study high treatment failure of AS + SP was observed at all the three sites ranging between 17 and 23%. A recent study conducted in the neighbouring area of northeast also showed AS + SP treatment failure of 9.5% [25]. This indicates that the reported treatment failure of AS + SP is frequent and widespread as the sites were present in four distant corners of the region and thus is likely to be present in between areas as well. The treatment failures reported in the study could be due to resistance to partner drug SP as there has been long history of use and documented SP failure in the region [5]. Also, irrational treatment practices as well as non-availability of age-based combi blister packs for children might have caused drug pressure in the population leading to failure of partner drug [26,27]. However, combination therapy with ineffective partner drug is equivalent to artemisinin monotherapy on a large scale.

Until recently, the efficacy and safety of recommended ACT (AS + SP) was high at multiple sites in the country [28]. The nationwide sentinel site monitoring system conducted 40 studies up to 2011 and the efficacy of AS + SP during 28 days’ follow-up was above WHO recommended cut-off for treatment failure at all the sites. The nationwide sentinel site system also monitored partner drug resistance through molecular studies given the long half life of SP to detect emerging resistance. Despite low prevalence of treatment failures to AS + SP, molecular markers of partner drug resistance were monitored to capture early signs of treatment failure. Emerging increasing trend of partner drug resistance markers and to capture LTF, if any, follow-up in the sentinel site monitoring system was extended to 42 days since 2012. Until 2010, *dhps* remained wild in majority of the samples, however, increasing trend in *dhps* point mutations were observed later, particularly in northeastern region. These findings prompted more focus in northeastern region. Thus, three sites at different ecological conditions were selected and efficacy of AS + SP in *P. falciparum* was studied.

Data on molecular studies for SP resistance are conclusive with increased frequency of quintuple mutation over time. Increased frequency of quadruple mutation in *dhfr* and *dhps* gene was observed from all the three study sites with higher frequency in *dhps* gene. Sulphadoxine resistance in *P. falciparum* is associated with mutations at five *pfdhps* codons; 436ala/phe, 437gly, 540glu, 581gly, and 613ser [10]. The *pfdhps* mutation at codon A436 causes alteration in binding of sulphadoxine followed by sequential mutations at G437, E540, G581, and T/S613 which may cause increase in sulphadoxine resistance [29]. Mutation at codon G581 along with 437 and 540 may be associated with earlier treatment failure [30].

A strong indicator for SP treatment failure is the quintuple mutations in three *pfdhfr* codons (108asn + 51ile + 59arg) and two *pfdhps* codons (437gly + 540glu) [31]. Quintuple mutation has been observed in 39% of treatment failure samples at all the three sites with highest in Tripura, confirming the role of SP failure. In the present study, quintuple as well as more combination of mutations have been observed at all the three sites with highest in Tripura. Similarly, high prevalence of quintuple mutation (35%) has been observed in samples collected from Kolkata [25].

Following these findings, further new studies on tracking resistance to artemisinin are being undertaken at two sites in this region. Recently association of K13-propeller polymorphism with artemisinin resistance *in vitro* and *in vivo* has been proposed [32]. Further studies to map K13 gene polymorphism, in addition to the clinical phenotype of slow clearance, are being undertaken.

Various factors including host immunity and pharmacokinetics of the drug play an important role in achieving complete cure or treatment failure in patients. In the present study, younger age category (<five years) relative to adults 15 years or older, fever at enrolment and low administered dose of AS (< 3 mg/kg, 3–3.5 mg/kg) relative to 3.5 mg/kg or more were associated with higher risk of treatment failure. Negative correlation was observed between the risk of failure with the dose of AS (in mg/kg body weight). Although, the recommended daily dose of AS is 4 mg/kg, 15.6% of treatment failure patients received 3.0 to < 3.5 mg of AS per kg, and 9.4% received < 3.0 mg/kg. Due to variation in body weight within age categories, dosing of drugs based on age category presents the risk of under-dosing or over-dosing. Thus, the criteria to use age rather than body weight for calculating the dose of anti-malarial needed for a patient is the probable cause of suboptimal dosing. However, optimal or higher dose of SP was observed in majority of the patients with only 9.4% patients receiving under-dose of SP. Fever at enrolment was positively and significantly associated with treatment failures. Earlier results of nation-wide sentinel system also observed younger age, fever and low AS dose as the potential risk factors for treatment failure [28]. Partner drug resistance markers are well defined and can ascertain the falling efficacy of the administered drug. The relative risk was highest with the presence of triple or quadruple mutation, which signifies the role of SP in treatment failure. Parasite clearance time is multifactorial and is affected by initial parasite load, host immunity, pharmacokinetics of the drug. No significant difference in mean parasitaemia, dose of AS at enrolment or any adverse event reported was observed between the patients who had parasitaemia ≥ 72 hours with those who cleared parasite within 48 hours. Host immunity had a definite role as six out of eight patients were less than five years of age. Also, poor absorption of drug in individuals may play a role in delayed PCT besides immunity. However, there were insufficient observations to determine the risk factors for parasite clearance interval of ≥ 72 hours.

Recently increasing failure rates of artesunate mefloquine in Thailand and dihydroartemisinin piperaquine in Cambodia have been reported [16,33]. With increasing cases of ACT failure, more studies to optimize the dose and duration of recommended ACT or new combination is required. The newly recommended ACT: artemether-lumefantrine (AL) has been used by neighbouring countries in the past; hence cross-border monitoring of AL with countries that use this regimen is urgently required. In addition, the newly introduced ACT in the region has certain challenges as the compliance to twice daily regimen and the fat dependant absorption in diet need to be considered besides the molecular markers for lumefantrine, as well as other partner drugs. Expanded *in vitro* monitoring in the northeast for early warning of this ACT is required.

## Conclusions

The efficacy of AS + SP was declining in northeast India and exceeded the threshold for changing drug policy. Based on the results of the study, the expert committee of the National Vector Borne Disease Control Programme formulated the first subnational drug policy for India and selected artemether plus lumefantrine as the new first-line treatment in the northeast. Continued monitoring of anti-malarial drug efficacy in northeastern region is essential for effective malaria control.

### Limitations

The nation-wide sentinel site monitoring system created in 2009 accelerated the assessment of emerging ACT treatment failure in areas with international borders and is vigilant to detect delayed parasite clearance, if any. Few of the earlier reported limitations of nationwide sentinel system were overcome during 2012. Firstly, follow-up was extended up to 42 days, delayed parasitaemia was monitored by looking at parasite clearance interval of ≥ 72 hours; partner drug resistance markers were analysed in all the isolates. The other limitations include 24 hours sampling for parasite monitoring, as the patients were from distant places. The sample size was relatively low in Changlang district, Arunachal Pradesh. To ascertain the role of partner drug resistance, drug levels of SP could not be done as the samples for drug concentration could not be collected.

## Competing interests

The authors declare that they have no competing interests.

## Authors’ contribution

NM, NV and GSS were responsible for the design of the project proposal and monitored progress. NM, KK, BS, JPN and RSB were involved in quality check of data and molecular studies. NM, VD and SP monitored the studies at the site. NM, RR and NKS compiled and performed statistical analysis. NM and NKS wrote the first draft and ACD, GSS, ARA and NV corrected the draft and all authors read and approved the final manuscript.
